# SES and Multimorbidity Among Black Women and Men: A Case of Diminishing Returns?

**DOI:** 10.1093/geroni/igaf122.1823

**Published:** 2025-12-31

**Authors:** Millicent Robinson, Courtney Thomas Tobin, Xumeng Yan, Christy Erving, Hans Oh, Angela Gutierrez

**Affiliations:** The University of North Carolina at Chapel Hill, Chapel Hill, North Carolina, United States; University of California, Los Angeles Fielding School of Public Health, Los Angeles, California, United States; University of California, Los Angeles Fielding School of Public Health, Los Angeles, California, United States; The University of Texas at Austin, Austin, Texas, United States; University of Southern California, Los Angeles, California, United States; Western University of Health Sciences, Pomona, California, United States

## Abstract

Higher socioeconomic status (SES) is typically associated with better health, but research suggests that Black Americans do not experience the same health benefits from SES as other groups. While multimorbidity (MM) is a growing public health concern, few studies have examined how SES influences MM among Black Americans. Using data from the Nashville Stress and Health Study, we investigated the relationship between several SES indicators—education, household income, occupational prestige, and a combined SES index—and MM among Black women (n = 325) and men (n = 296). Full sample and gender-stratified negative binomial regression models indicate limited associations between SES and MM, with effects primarily observed among men. Among women, no significant associations between SES and MM were found. Among men, having some college education was linked to higher MM (IRR=1.50, 95% CI: 1.06-2.12, p = 0.024), while a bachelor’s degree or higher was protective (IRR=0.58, 95% CI: 0.38-0.89, p = 0.013). Middle-income men had lower MM (IRR=0.66, 95% CI: 0.46-0.96, p = 0.031), and occupational prestige was inversely associated with MM (IRR=0.99, 95% CI: 0.99-1.00, p = 0.022). These findings support the diminishing returns hypothesis, demonstrating that SES does not uniformly translate to better health for Black Americans, particularly Black women. The absence of significant associations for women highlights the need for further research on gendered SES-health relationships to address MM inequities in this population.

